# Internalization Mechanisms of the Epidermal Growth Factor Receptor after Activation with Different Ligands

**DOI:** 10.1371/journal.pone.0058148

**Published:** 2013-03-05

**Authors:** Lasse Henriksen, Michael Vibo Grandal, Stine Louise Jeppe Knudsen, Bo van Deurs, Lene Melsæther Grøvdal

**Affiliations:** Department of Cellular and Molecular Medicine, University of Copenhagen, Copenhagen, Denmark; Institut Curie, France

## Abstract

The epidermal growth factor receptor (EGFR) regulates normal growth and differentiation, but dysregulation of the receptor or one of the EGFR ligands is involved in the pathogenesis of many cancers. There are eight ligands for EGFR, however most of the research into trafficking of the receptor after ligand activation focuses on the effect of epidermal growth factor (EGF) and transforming growth factor-α (TGF-α). For a long time it was believed that clathrin-mediated endocytosis was the major pathway for internalization of the receptor, but recent work suggests that different pathways exist. Here we show that clathrin ablation completely inhibits internalization of EGF- and TGF-α-stimulated receptor, however the inhibition of receptor internalization in cells treated with heparin-binding EGF-like growth factor (HB-EGF) or betacellulin (BTC) was only partial. In contrast, clathrin knockdown fully inhibits EGFR degradation after all ligands tested. Furthermore, inhibition of dynamin function blocked EGFR internalization after stimulation with all ligands. Knocking out a number of clathrin-independent dynamin-dependent pathways of internalization had no effect on the ligand-induced endocytosis of the EGFR. We suggest that EGF and TGF-α lead to EGFR endocytosis mainly via the clathrin-mediated pathway. Furthermore, we suggest that HB-EGF and BTC also lead to EGFR endocytosis via a clathrin-mediated pathway, but can additionally use an unidentified internalization pathway or better recruit the small amount of clathrin remaining after clathrin knockdown.

## Introduction

The epidermal growth factor receptor (EGFR), a receptor tyrosine kinase (RTK), is a member of the ErbB family of signaling receptors. It is involved in regulating growth and differentiation as well as in the pathogenesis of a number of cancers.

The EGFR is activated upon ligand binding. Activation leads to internalization of the receptor and trafficking to the early endosomal compartment of the cell. From there the receptor can either be degraded via lysosomes or recycled to the cell surface depending on the ligand bound (reviewed in [1], [2]. Endocytic downregulation of the EGFR is an important mechanism of signal attenuation.

So far eight ligands for EGFR have been described. These are epidermal growth factor (EGF), transforming growth factor-*α* (TGF-*α*), heparin-binding EGF-like growth factor (HB-EGF), betacellulin (BTC), amphiregulin (AR), epiregulin (EPI), epigen and neuregulin2-*β* [3], [4].

Although EGFR is one of the most thoroughly studied RTKs, little is known about the different roles of the EGFR ligands. Until recently most of the studies on EGFR trafficking were performed using EGF-stimulation and a few using TGF-*α*-stimulation. These studies showed a difference in trafficking, where EGF leads to receptor degradation whereas TGF-*α* leads to recycling of the receptor [5]. This is caused by a difference in pH sensitivity, causing dissociation of TGF-*α* from EGFR in endosomes. EGF however, remains bound to EGFR [6].

Earlier we have described how EGFR endocytosis is differentially regulated by the various ligands [7]. EGF causes an intermediary amount of receptor internalization and degradation with some recycling of the receptor. HB-EGF- and BTC-stimulation lead to a strong induction of internalization and to degradation of a large portion of the internalized receptor. TGF-*α* and EPI lead to an intermediate internalization but an almost complete recycling. AR stimulation leads to receptor internalization, but the receptor did not readily return to the surface, possibly because of recycling via a slow recycling pathway.

Until recently ligand-induced EGFR endocytosis was believed to be primarily clathrin-mediated. It has been reported that the ligand-induced receptor internalization has a relatively low capacity, thus the pathway is easily saturated [8], [9]. In 2003 Hinrichsen and colleagues found that clathrin-knockdown with siRNA does not block EGFR uptake in cells [10]. Recently it was reported that the EGFR can be internalized by macropinocytosis in certain cell types [11]–[13]. Sigismund and colleagues reported that a clathrin-independent mechanism of endocytosis exists for the EGFR. They found that this endocytosis occurs after stimulation with high concentrations of EGF [14], [15]. In contrast, results from our own group and collaborators indicate that EGF-stimulated receptor endocytosis can be completely abolished with clathrin knockdown [16], [17].

Here we have investigated the internalization mechanisms of EGFR after addition of different ligands and inhibition of several endocytic pathways. Endocytosis of EGFR after treatment with all ligands could be inhibited to a certain degree by ablation of clathrin. However, after HB-EGF or BTC stimulation, internalization was not fully inhibited. Additionally we found that inhibition of dynamin function blocked internalization after stimulation with all ligands. However, inhibition of macropinocytosis, caveolin1, RhoA, Arf6, flotillin1/2 or raft-dependent pathways did not have an effect on internalization of the EGFR. These data suggest that the more potent ligands HB-EGF and BTC could induce a so far unknown pathway of internalization or they could be better at utilizing the small amounts of clathrin remaining after knockdown.

## Results

### Clathrin Knockdown Fully Inhibits EGF-induced EGFR Internalization, but Internalization Upon HB-EGF and BTC Binding is Only Partly Inhibited

**Figure 1:**
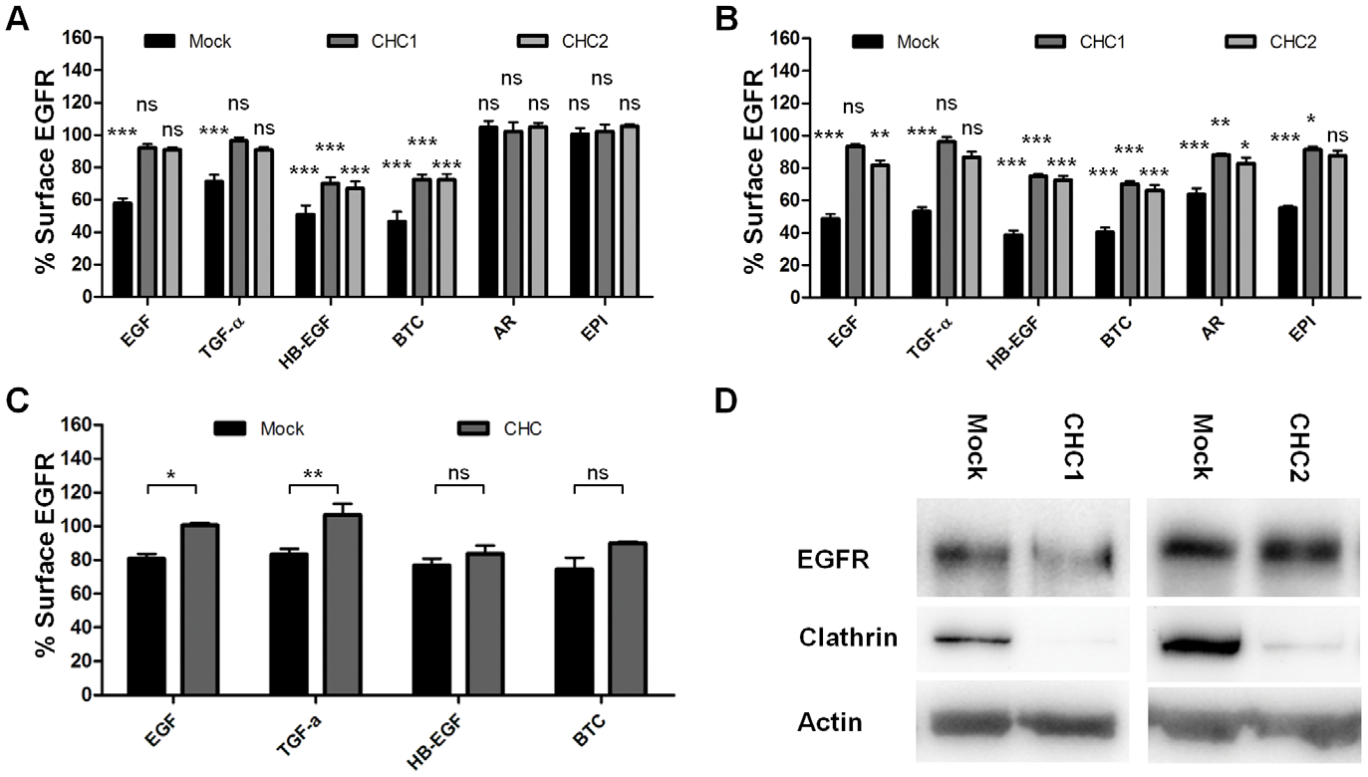
EGFR internalization after clathrin knockdown. A: Cells treated with siRNA were incubated with 3.22 nM ligand for 15 minutes at 37°C. The amount of cell surface EGFR was determined by flow cytometry and data normalized to unstimulated cells. Data points represent mean+SEM. Statistical analysis comparing each column to their relevant unstimulated control (normalized to 100) was performed using one-way ANOVA with Bonferroni posttest. *** = p<0.001, ns = non significant. B: Knockdown cells were incubated with 10 nM (EGF, TGF-α, HB-EGF, BTC) or 100 nM (AR, EPI) ligand for 15 minutes at 37°C. The amount of cell surface EGFR was determined by flow cytometry and data normalized to unstimulated cells. Data points represent mean+SEM. Statistical analysis comparing each column to their relevant unstimulated control (normalized to 100) was performed using one-way ANOVA with Bonferroni posttest. * = p<0.05, ** = p<0.01, *** = p<0.001, ns = non significant. C: Knockdown cells were incubated with 10 nM ligand for 5 minutes at 37°C. The amount of cell surface EGFR was determined by flow cytometry and data normalized to unstimulated cells. Data points represent mean+SEM. Statistical analysis comparing CHC to mock-treatment for each ligand was performed using two-way ANOVA with Bonferroni posttest. * = p<0.05, ** = p<0.01, ns = non significant. D: Test of the clathrin knockdown and EGFR levels. Cells were lysed in RIPA buffer and resolved by SDS-PAGE and western blotting. Actin is used as a loading control.

To determine the dependency of EGFR internalization on clathrin after stimulation with the different ligands, the amount of cell surface receptor was measured after clathrin knockdown and addition of ligands. HeLa cells were treated with siRNA against clathrin heavy chain (CHC), and stimulated with 3.22 nM ligand for 15 minutes. For EGF this corresponds to 20 ng/ml, a concentration reported to induce both clathrin-mediated and clathrin-independent endocytosis of EGFR [14]. In cells treated with EGF or TGF-α, knockdown using either of 2 CHC siRNA sequences returned surface receptor levels to levels similar to those in unstimulated cells (Figure 1A). In contrast, HB-EGF- and BTC-induced internalization was only partly inhibited by clathrin knockdown. In cells treated with clathrin siRNA and these ligands, surface EGFR levels were only increased to approximately 70% of surface levels in unstimulated cells. 3.22 nM AR or EPI did not induce significant EGFR internalization.

To further test for clathrin-dependency of ligand-induced internalization, we stimulated clathrin knockdown cells with concentrations of ligand previously shown to induce maximum levels of internalization [7]. HeLa cells were treated with 10 nM (EGF, TGF-*α*, HB-EGF and BTC) or 100 nM ligand (AR and EPI) for 15 minutes. As seen in Figure 1B, the effects of clathrin siRNA, were similar to those observed at lower ligand concentrations (see Figure 1A). CHC knockdown using one siRNA sequence (CHC1) blocked EGFR endocytosis, returning the surface receptor levels to those in unstimulated cells for EGF- and TGF-*α*-treated cells. The second siRNA sequence (CHC2) was not as potent at this concentration, and therefore not used in further experiments. Thus, CHC1 siRNA is henceforth referred to as CHC siRNA. Stimulation with 100 nM AR or EPI led to receptor internalization and this internalization was significantly inhibited by clathrin knockdown. Receptor levels on the surface of cells treated with HB-EGF or BTC were also in this case only about 70% of those on unstimulated cells. As AR and EPI are weak inducers of EGFR internalization unless used at very high concentrations, and also strongly dependent on clathrin for internalization, we chose to focus on EGF, TGF-a, HB-EGF and BTC for further studies. To investigate if these differences in receptor surface levels could be due to differences in EGFR recycling, internalization was also studied after only 5 minutes of ligand stimulation, reducing the contribution of recycling (Figure 1C). After 5 minutes stimulation, internalization was significantly inhibited by clathrin siRNA for EGF and TGF-α. However, as observed at 15 minutes, inhibition was less efficient for HB-EGF and BTC stimulation. Combined, these results indicate that although clathrin knockdown fully inhibits internalization of EGFR after stimulation with EGF and TGF-*α*, a fraction of the receptors stimulated with HB-EGF or BTC is still internalized. Clathrin knockdown did not appear to affect EGFR levels (Figure 1D).

### Clathrin Knockdown Abolishes EGF-induced EGFR Localization to Endosomes, Whereas HB-EGF- and BTC-induced Endosomal Localization is Only Partly Reduced

**Figure 2:**
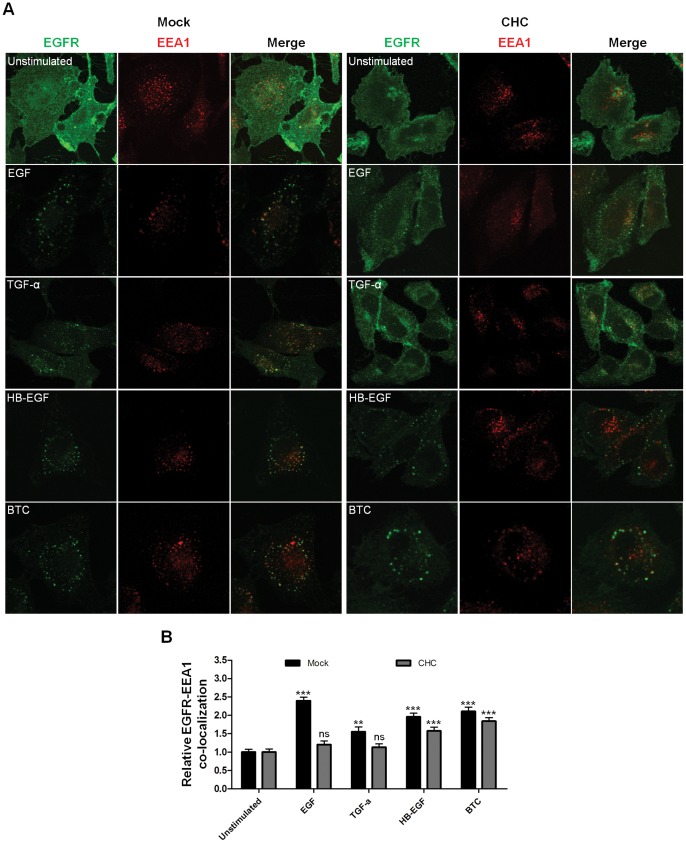
EGFR co-localization with EEA1 after clathrin knockdown. A: Cells treated with siRNA were incubated with 10 nM ligand for 15 minutes at 37°C. The cells were fixed and labeled for EGFR and EEA1. B: Quantitative analysis of the amount of EGFR co-localizing with EEA1 in an average of 32–51 cells for each ligand+SEM. Statistical analysis comparing each column to their relevant unstimulated control (normalized to 1) was performed using one-way ANOVA with Bonferroni posttest. ** = p<0.01, *** = p<0.001, ns = non significant.

To further study the clathrin-dependency of EGFR endocytosis, HeLa cells treated with siRNA against CHC were stimulated with 10 nM ligand for 15 minutes, as we have previously shown that maximum internalization is reached after 15 minutes stimulation with all ligands [7]. Cells were fixed and labeled for EGFR and the endosomal marker EEA1. As seen in Figure 2, clathrin knockdown largely abolished the co-localization between EGFR and EEA1 in cells stimulated with EGF or TGF-α, corresponding to the high EGFR surface levels observed in Figure 1. In the two lower panels in Figure 2A, it is seen that EGFR still co-localizes with EEA1 in cells treated with clathrin siRNA when stimulated with HB-EGF or BTC. Thus, clathrin knockdown using siRNA does not fully inhibit internalization of EGFR into early endosomes, upon binding of HB-EGF or BTC. This suggests that the remaining internalization in clathrin knockdown cells observed in Figure 1 leads to sorting of the EGFR to early endosomes.

### EGFR Degradation is Inhibited by Clathrin Knockdown

**Figure 3:**
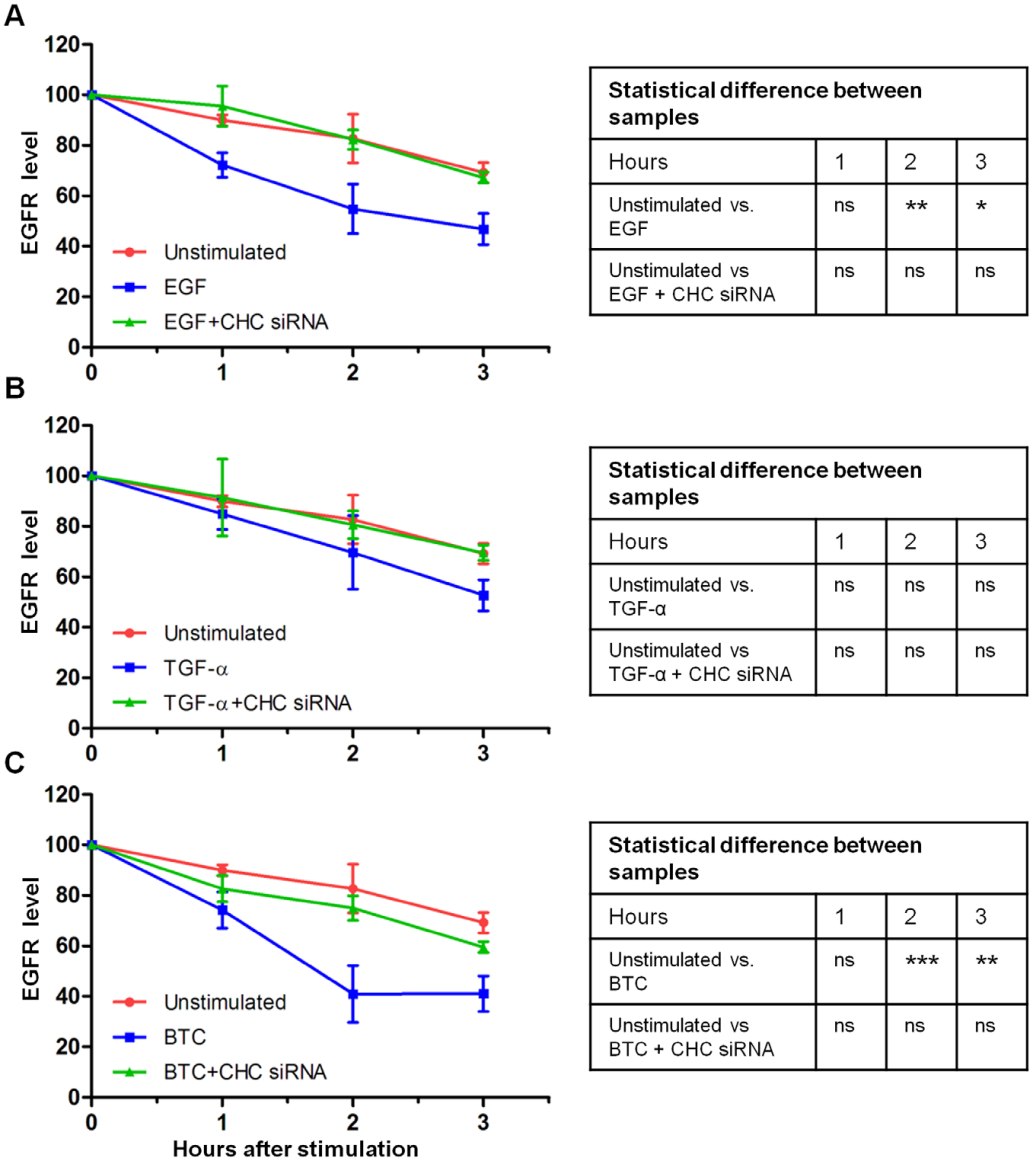
EGFR degradation after knockdown with CHC siRNA and ligand stimulation. Cells were incubated with 10 nM of the indicated ligand at 37°C for different time periods. Cells were lysed and the amount of EGFR determined by ELISA. Data points represent mean +/− SEM. A: EGF, B: TGF- α, C: BTC. Statistical analysis comparing degradation in unstimulated cells to ligand-treated and ligand+CHC siRNA-treated cells was performed using two-way ANOVA with Bonferroni posttest. * = p<0.05, ** = p<0.01, *** = p<0.001, ns = non significant.

It has previously been reported that different mechanisms of internalization of the EGFR can lead to differences in receptor degradation [14]. Also, our earlier work has shown how different ligands led to a different fate for EGFR [7]. The observation that internalization of EGFR was differentially dependent on clathrin for the various EGFR ligands therefore led us to investigate how degradation of the EGFR is dependent on clathrin-mediated endocytosis after stimulation with the EGFR ligands. HeLa cells were treated with siRNA targeting CHC and stimulated with EGF, TGF-*α* or BTC for 0–3 hours. The cells were then lysed and EGFR levels were measured by ELISA. Figure 3 shows EGFR levels in cells treated with 10 nM ligand. Knockdown of clathrin inhibited degradation of EGFR after stimulation with EGF (Figure 3A). TGF-α did not induce significant EGFR degradation (Figure 3B). When cells were stimulated with BTC (Figure 3C) degradation was also strongly inhibited by clathrin siRNA. Thus degradation of EGFR after addition of ligand seems to be dependent on clathrin for all 3 ligands. This indicates that the internalization of BTC-stimulated EGFR in clathrin knockdown cells leads to sorting to early endosomes. However, here the receptor is no longer sorted for degradation. We therefore set out to investigate if another mechanism of internalization could be involved in EGFR endocytosis upon stimulation with HB-EGF and BTC.

### The Macropinocytosis Inhibitor Amiloride does not Inhibit EGFR Internalization

**Figure 4:**
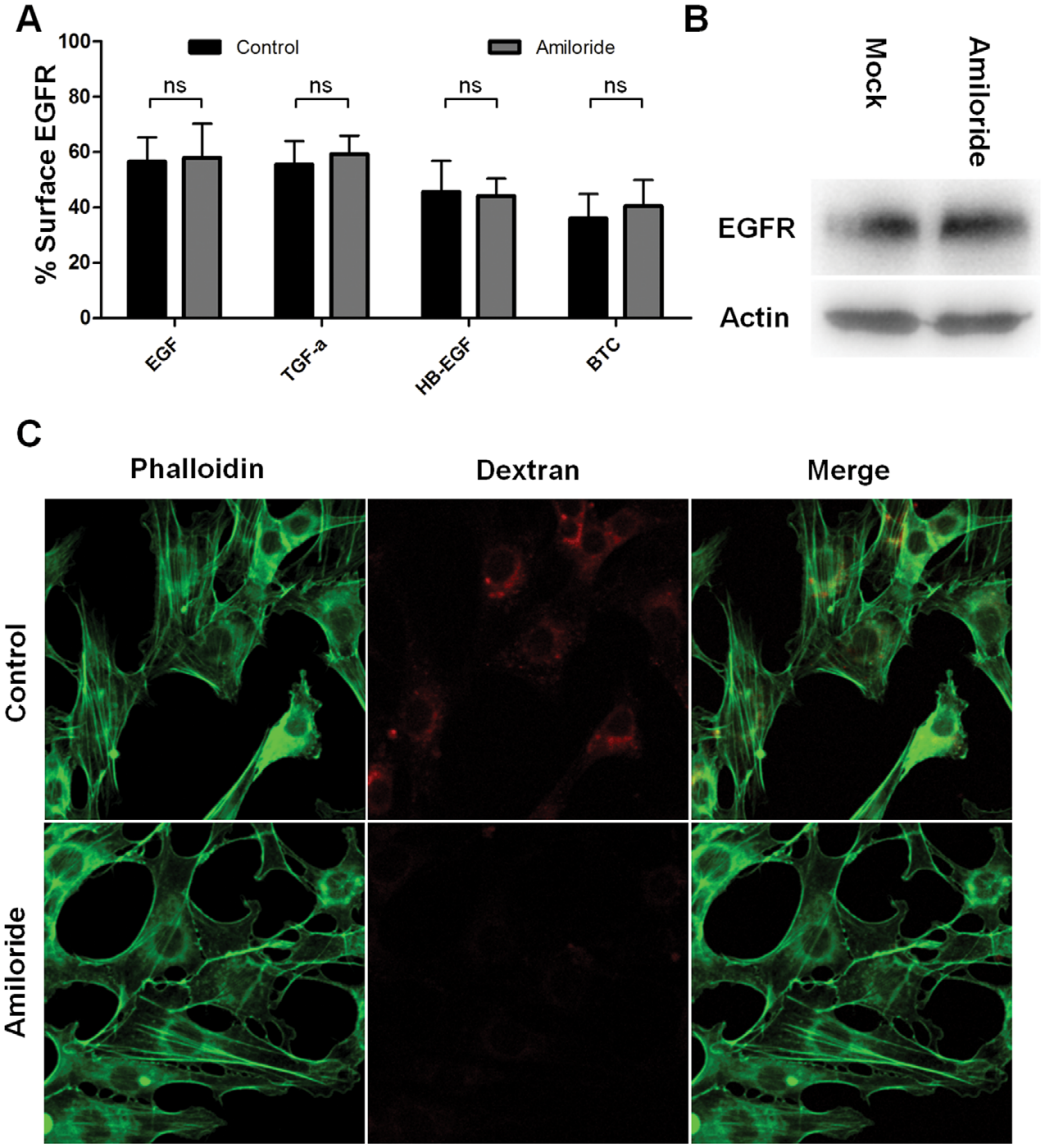
EGFR internalization after treatment with amiloride. A: Cells were incubated with or without 1 mM amiloride 15 minutes prior to stimulation with ligand. After the incubation cells were treated with 10 nM EGFR ligand for 15 minutes at 37°C. The amount of cell surface EGFR was determined by flow cytometry and data normalized to unstimulated cells. Data points represent mean+SEM. Statistical analysis comparing amiloride to control treatment for each ligand was performed using two-way ANOVA with Bonferroni posttest. ns = non significant. B: Test of EGFR levels. Cells were lysed in RIPA buffer and resolved by SDS-PAGE and western blotting. Actin is used as a loading control. C: NIH-3T3 cells in 8-chamber culture wells were challenged with 250 ug/ml 70 kDa lysine-fixable Dextran-alexa488, with or without 1 mM amiloride in full growth medium for one hour before washing and fixation.

It has been reported that EGF concentrations of 100–200 ng/ml (corresponding to 16.1–32.2 nM) can induce macropinocytosis in A431 and MCF7 cells [12], [13], and that EGF treatment induces EGFR uptake by circular ruffling in NR6, COS7 and PANC1 cells [11]. To investigate if HB-EGF and BTC could induce EGFR internalization via macropinocytosis in HeLa cells, we used the macropinocytosis inhibitor amiloride [12]. Cells were incubated with 1 mM amiloride before stimulation with 10 nM of EGF, TGF-*α*, HB-EGF or BTC for 15 minutes. Cell surface receptor levels were determined by flow cytometry. Figure 4A shows that amiloride has no effect on EGFR receptor internalization for either of the ligands, indicating that HB-EGF or BTC-stimulation does not induce receptor internalization by macropinocytosis in HeLa cells. EGFR levels did not appear to be affected by amilorid treatment (Figure 4B). In Figure 4C it can be seen that amiloride inhibits uptake of a fluorescent dextran into NIH-3T3 cells demonstrating that the amiloride indeed inhibits macropinocytosis.

### EGFR Internalization after Ligand Binding is Dependent on Dynamin for All Ligands

**Figure 5:**
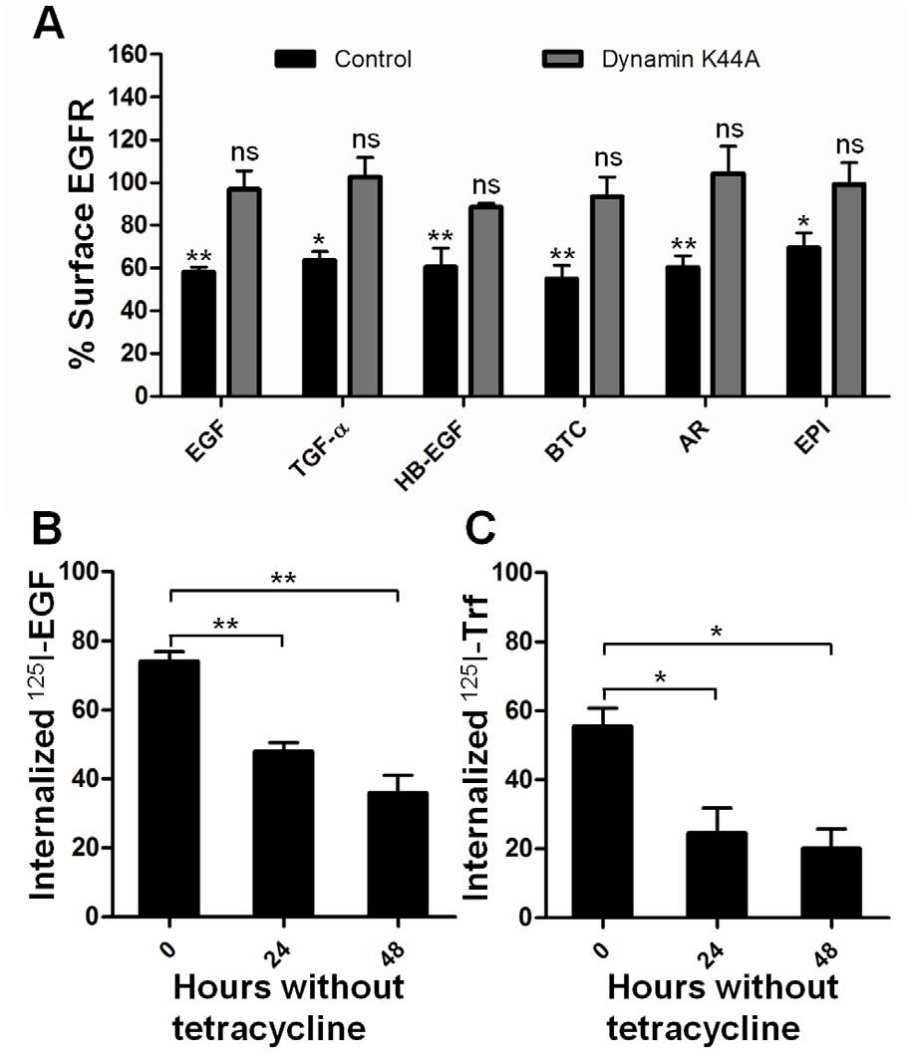
EGFR internalization in the HeLa dynamin K44A cell line. A: K44A dynamin expression was induced by removal of tetracycline from the medium for 48 hours. Cells were incubated with 10 nM (EGF, TGF-α, HB-EGF, BTC) or 100 nM (AR, EPI) ligand for 15 minutes at 37°C. The amount of cell surface EGFR was determined by flow cytometry and data normalized to unstimulated cells. Data points represent mean+SEM. Statistical analysis comparing each column to their relevant unstimulated control (normalized to 100) was performed using one-way ANOVA with Bonferroni posttest. * = p<0.05, ** = p<0.01, ns = non significant. B: K44A dynamin expression was induced by removal of tetracycline from the medium for 24 or 48 hours. Cells were incubated with ^125^I-EGF for 15 minutes at 37°C, the ligand bound on the surface was separated from the internalized. Data points represent mean+SEM. Statistical analysis comparing 0 hours to 24/48 hours was performed using one-way ANOVA with Bonferroni posttest. ** = p<0.01. C: K44A dynamin expression was induced by removal of tetracycline from the medium for 24 or 48 hours. Cells were incubated with ^125^I-transferrin for 15 minutes at 37°C, and then Pronase E for 45 minutes on ice to separate the surface bound ligand from the cells from the internalized fraction. Data points represent mean+SEM. Statistical analysis comparing 0 hours to 24/48 hours was performed using one-way ANOVA with Bonferroni posttest. * = p<0.05.

Having seen that macropinocytosis does not appear to be involved in HB-EGF- or BTC-induced internalization, we wanted to investigate how the internalization depends on dynamin. For this purpose, HeLa cells stably transfected with the dynamin K44A dominant negative mutant in an inducible tet-off system were grown without tetracycline for 48 hours to induce expression of the dynamin mutant. The cells were then treated with ligand and the cell surface EGFR levels were determined using flow cytometry. Figure 5A shows that expression of the dominant negative dynamin returns surface EGFR levels to that of unstimulated cells for all ligands, indicating that endocytosis of the receptor is dynamin-dependent regardless of ligand. Figure 5B+C shows that internalization of both radiolabeled transferrin and EGF is inhibited after 48 hours of dynamin K44A induction.

### Cholesterol-interference with the Drug Filipin does not Inhibit EGFR Internalization

**Figure 6:**
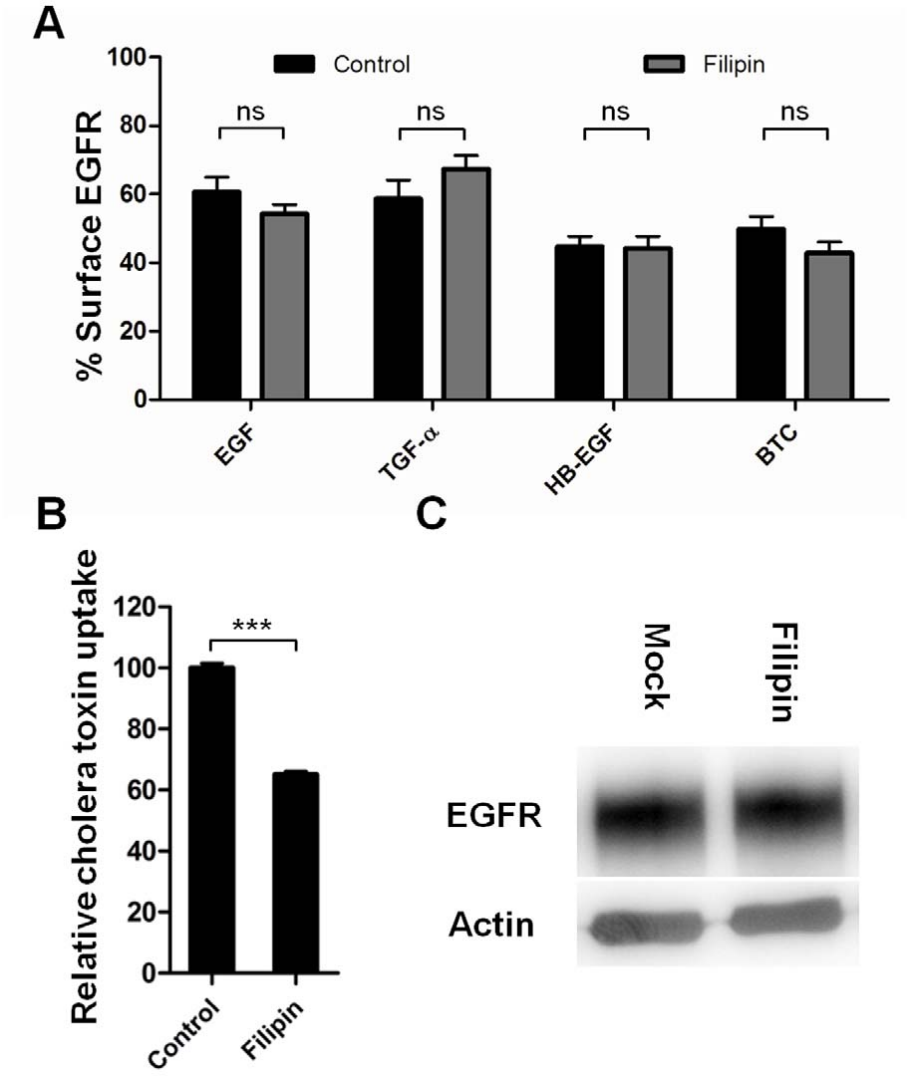
EGFR internalization after filipin treatment. A: Cells were incubated with or without 1 µg/ml filipin for 1 hour, and then treated with 10 nM ligand for 15 minutes at 37°C. The amount of cell surface EGFR was determined by flow cytometry and data normalized to unstimulated cells. Data points represent mean+SEM. Statistical analysis comparing filipin to control treatment for each ligand was performed using two-way ANOVA with Bonferroni posttest. ns = non significant. B: Cells were incubated with or without 1 µg/ml filipin for 1 hour, and then allowed to bind fluorescently labeled cholera toxin on ice for 30 minutes. The cholera toxin solution was removed and the cells were allowed to internalize the cholera toxin for 1 hour at 37°C. After uptake the cells were washed and cholera toxin uptake was determined using flow cytometry. Statistical analysis comparing filipin to control treatment was performed using t-test. *** = p<0.001. C: Test of EGFR levels. Cells were lysed in RIPA buffer and resolved by SDS-PAGE and western blotting. Actin is used as a loading control.

As many clathrin-independent pathways of endocytosis involve the presence of rafts [18], [19] and interference with cholesterol has been reported to inhibit EGFR internalization [14], we investigated if treatment with the cholesterol-sequestering drug filipin could inhibit receptor internalization after HB-EGF and BTC stimulation. HeLa cells were treated with 1 µg/ml filipin 1 hour prior to stimulation with 10 nM ligand for 15 minutes. Remaining receptors on the cell surface were measured using flow cytometry. Figure 6A shows that filipin does not inhibit EGFR internalization after ligand stimulation, thus HB-EGF and BTC-stimulation does not lead to cholesterol-dependent internalization of the EGFR. Figure 6B shows uptake of fluorescently labeled cholera toxin into filipin treated cells relative to control cells, showing that the filipin inhibits raft-mediated endocytosis. Filipin treatment did not appear to affect EGFR levels (Figure 6C).

### Knockdown of Caveolin1 does not Inhibit EGFR Internalization

To further investigate for the involvement of a possible clathrin-independent but dynamin-dependent mechanism in endocytosis of the EGFR after binding of HB-EGF or BTC, we looked at caveolae. It has been demonstrated that EGFR co-localizes with caveolin after stimulation with EGF [15]. Similarly, co-localization is seen between the TGF-β receptor and caveolin [20]. It has also been shown that when loaded with virus particles, caveolae can be downregulated in a dynamin-dependent manner [21], [22]. To investigate if HB-EGF and BTC stimulation induces caveolin-dependent receptor endocytosis, caveolin1 was ablated in HeLa cells, using siRNA.

**Figure 7:**
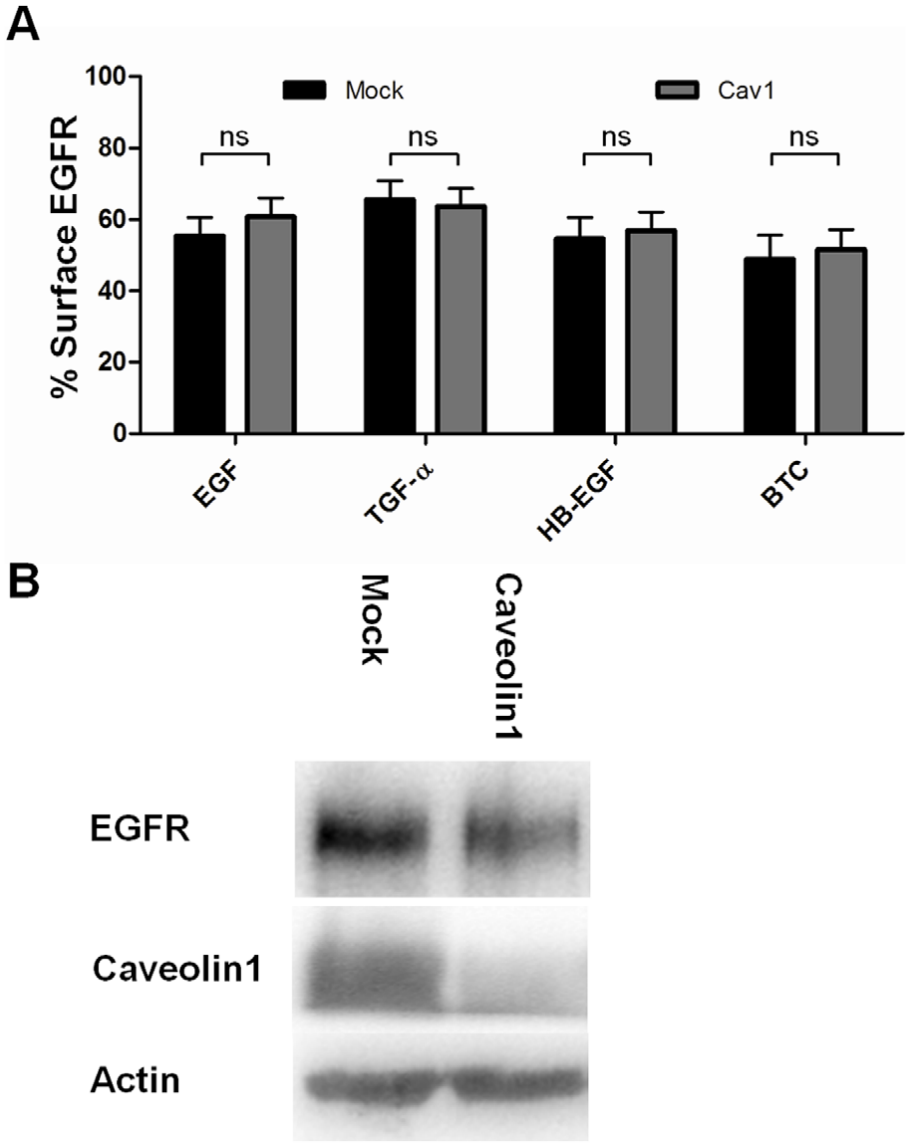
EGFR internalization after caveolin1 knockdown. A: Cells treated with siRNA were incubated with 10 nM ligand for 15 minutes at 37°C. The amount of cell surface EGFR was determined by flow cytometry and data normalized to unstimulated cells. Data points represent mean+SEM. Statistical analysis comparing caveolin siRNA treated cells to mock treatment for each ligand was performed using two-way ANOVA with Bonferroni posttest. ns = non significant. B: Test of the caveolin1 knockdown and EGFR levels. Cells were lysed in RIPA buffer and resolved on SDS-PAGE and western blotting. Actin is used as a loading control.

Cells were then stimulated with 10 nM EGF, TGF-α, HB-EGF or BTC for 15 minutes. Cell surface receptor levels were measured by flow cytometry. Knockdown of caveolin1 had no effect on the cell surface levels of EGFR after ligand stimulation (Figure 7), thus indicating that the protein is not involved in BTC- or HB-EGF-induced EGFR endocytosis. Knockdown of caveolin1 did not appear to affect EGFR levels (Figure 7B).

## Discussion

The internalization of EGFR has until recently been considered a textbook example of clathrin-dependent endocytosis, with receptor phosphorylation driving the internalization. Endocytosis of the receptor was considered to be purely a mechanism for attenuating signaling. Recent studies have suggested that EGFR endocytosis might not be as straightforward as was first believed, with the presence of many mechanisms of EGFR endocytosis, and the need for internalization for full activation of signaling pathways [11], [14], [15], [23], [24]. These studies, combined with our knowledge of the differential intracellular trafficking of EGFR after stimulation with its different ligands [7], stresses the need for a better understanding of how the different ligands affect EGFR internalization, sorting and signaling. In this study we found that ligand-induced EGFR internalization in our system was dependent on clathrin, but to a different degree depending on the ligand used (Figure 1). EGF-, TGF-*α-*, AR- and EPI-induced internalization was almost completely inhibited by clathrin knockdown, whereas HB-EGF- and BTC-induced internalization was only partly inhibited.

The observed dependency of EGFR endocytosis on clathrin upon stimulation with EGF is in agreement with the earlier findings of our group and collaborators [16], but in contrast to the results of others [10], [14], [15]. These studies all use HeLa cells, but the different results could very well be the result of clonal differences: Differences in the expression levels of proteins involved in EGFR internalization could contribute to the differences in internalization pathways used between cell lines. In fact, gene expression analysis have revealed large differences in the gene expression profiles of the HeLa cell lines used in these studies (personal communication, Pier Paolo Di Fiore).

We found that HB-EGF- and BTC-induced EGFR internalization could not be fully inhibited by clathrin knockdown (Figure 1). This could indicate that HB-EGF- and BTC-stimulation induce internalization via a clathrin-independent pathway. As BTC and HB-EGF are more potent inducers of EGFR activation than EGF [7], this mechanism could be the same as that reported by others at high concentrations of EGF. However, Sigismund and colleagues found that the cholesterol-interfering drug filipin can inhibit endocytosis of EGFR after stimulation with medium and high concentrations of EGF [14]. We did not observe any effect of filipin on HB-EGF- and BTC-induced endocytosis (Figure 6). Thus, the same mechanisms do not appear to be involved. We were not able to positively identify an alternative mechanism of EGFR internalization, despite inhibiting a range of known mediators of internalization (Figure 4+6–7, Figures S1, S2, S3). Although macropinocytosis is a known pathway for EGFR internalization [11]–[13] we saw no effect on the uptake of EGFR after ligand-stimulation in our cells (Figure 4). Similarly we found no effect of inhibiting caveolin1 (Figure 7), RhoA, Arf6 or flotillin1/2 (Figures S1, S2, S3) on EGFR internalization after stimulation with different ligands. This could indicate the involvement of an internalization pathway that has yet to be characterized. Also, long-term inhibition of endocytosis, such as here, can potentially lead to cellular stress, which in turn can lead to upregulation of alternative mechanisms of endocytosis [25].

In this study we have utilized siRNA to inhibit clathrin-mediated endocytosis. Although this is a potent tool for inhibiting protein expression, the clathrin protein knockdown is not 100% efficient. Thus we do observe small amount of remaining clathrin protein by western blotting (∼5%) upon siRNA treatment. The remaining clathrin levels might be high enough to support the observed EGFR endocytosis if the receptor can actively recruit the remaining clathrin. This would correlate with the fact that the two ligands that can overcome the inhibiting effects of clathrin siRNA, HB-EGF and BTC, are also the two most potent activators of EGFR [7], and the reported observation that activation of EGFR can lead to formation of new clathrin-coated pits [26].

In our earlier work we have shown that HB-EGF and BTC are potent inducers of receptor degradation [7]. To determine if the internalization observed after stimulation with HB-EGF or BTC stimulation led to degradation we measured degradation in cells after clathrin inhibition. We found that knocking down clathrin led to an almost complete inhibition of receptor degradation (Figure 3). This could indicate the presence of an alternative mechanism of internalization that does not lead to degradation. However, clathrin is also involved in endosomal sorting for degradation, and it is therefore possible that the observed inhibition in degradation of EGFR upon BTC stimulation in clathrin knockdown cells, in spite of continued internalization, could be due to impaired sorting machinery in these cells, as recruitment of the endosomal ESCRT machinery requires the presence of clathrin [27].

This study further supports the importance of clathrin in EGFR endocytosis. However, the fact that clathrin independent regulation of EGFR appears to be important in other cells suggests that EGFR can also utilize other pathways of endocytosis in cancer. Our results also show that internalization of the EGFR can differ depending on which ligand is bound, and further stress the importance of determining the identity of the ligands present in a tumor environment when studying and predicting EGFR signaling and regulation.

## Materials and Methods

Unless otherwise stated, reagents were purchased from Sigma-Aldrich, St. Louis, Missouri.

### Cell Culture

HeLa and HeLa Arf6 Q67L cells were kindly provided by Kirsten Sandvig (Centre for Cancer Biomedicine, University of Oslo). HeLa cells were maintained in DMEM supplemented with 10% FCS (Biosera, Ringmer, United Kingdom), 2 mM glutamax (Invitrogen, Carlsbad, California), 1 mM sodium pyruvate, 100 U/ml penicillin, and 100 µg/ml streptomycin. The HeLa Arf6 Q67L cells were maintained in DMEM supplemented with 10% FCS, 2 mM L-glutamine, 100 units ml^−1^ penicillin, 100 µg ml^−1^ streptomycin, 400 µg ml^−1^ geneticin, 50 µg ml^−1^ hygromycin B and 1 mg ml^−1^ tetracycline. Dynamin K44A HeLa cells were grown in DMEM supplemented with 10% FCS, 2 mM L-glutamine, 100 units ml^−1^ penicillin, 100 µg ml^−1^ streptomycin, 400 µg ml^−1^ geneticin, 200 ng ml^−1^ puromycin and 1 mg ml^−1^ tetracycline. This cell line [28] was kindly provided by Claudia Krag (BRIC, University of Copenhagen). NIH-3T3 cells were kindly provided by Frederik Vilhardt (Institute for Cellular and Molecular Medicine, University of Copenhagen). The NIH-3T3 cells were maintained in DMEM supplemented with 10% FCS, 2 mM glutamax and 100 units ml^−1^ penicillin, 100 mg ml^−1^ streptomycin. Cells were serum starved in starvation medium (DMEM supplemented with 2 mM glutamax, 1 mM sodium pyruvate, 100 U/ml penicillin, and 100 µg/ml streptomycin) 1–2 h prior to experiments.

### EGFR Internalization Analysis

Cells were incubated with ligand (RnD Systems, Minneapolis, Minnesota). Cells were washed with ice-cold PBS, washed 5 minutes with an ice-cold acidic buffer (100 mM NaCl, 50 mM glycine, pH 2.5) to remove any remaining bound ligand, neutralized with ice-cold PBS and trypsinized on ice until detachment. Trypsin was neutralized by addition of soy bean trypsin inhibitor, and the detached cells were fixed for 15 minutes in ice-cold 2% paraformaldehyde. The amount of EGFR present at the cell surface was determined by labeling of the unpermeabilized and fixed cells with an anti-EGFR antibody directly conjugated to FITC (AbD serotec, Oxford, UK) followed by flow cytometric analysis to quantify EGFR surface labeling.

### Protein Knockdown

HeLa cells were transfected twice with siRNA against clathrin heavy chain (sense sequence 1: GCAAUGAGCUGUUUGAAGA, [29] sense sequence 2: CCUGCGGUCUGGAGUCAAC [14]), or once with caveolin1 (sense sequence: UGUCUGGGGGCAAAUACG), flotillin1 (sense sequence GCAGAGAAGUCCCAACUAAUU [30]), flotillin2 (sense sequence: GAGGUUGUGCAGCGCAAUU [30]) or scrambled siRNA (mock) (AM4635, Ambion, Austin, Texas/Allstars negative siRNA, Qiagen) using Lipofectamine2000 (Invitrogen, Carlsbad, California).

Transfections with CHC siRNA were done with 48 hour intervals and experiments were performed 48 h after the second transfection. Cells were transfected once with caveolin1, flotillin1 or flotillin2 siRNA and experiments were performed 48 hours after transfection. Protein knockdown was evaluated by western blotting using a mouse monoclonal α-CHC antibody (Fitzgerald Antibodies, Concord, Massachusetts), a rabbit polyclonal caveolin1 antibody (Transduction laboratories, Lexington, Kentucky), a mouse monoclonal Arf6 antibody (Santa Cruz Biotechnology, California), a rabbit polyclonal flotillin1 antibody (Sigma-aldrich) a mouse monoclonal flotillin2 antibody (Transduction laboratories, Lexington, Kentucky). A mouse monoclonal actin antibody (Sigma-Aldrich) and sheep polyclonal anti-EGFR antibody (Fitzgerald Antibodies) were used as controls. HRP-conjugated secondary antibodies were from DAKO (Glostrup, Denmark).

### Inhibition with Amiloride

HeLa cells were incubated with 1 mM amiloride 15 minutes prior to stimulation with ligand. NIH-3T3 cells in 8-chamber culture wells were challenged with 250 ug/ml 70 kDa lysine-fixable dextran-alexa488, with or without 1 mM amiloride in full growth medium for one hour before washing and fixation and examined by microscopy.

### Inhibition with Filipin

HeLa cells were incubated with 1 µg/ml filipin 1 hour prior to stimulation with ligand. To determine filipin activity the ability to inhibit uptake of 0.2 µg/ml alexa488-conjugated cholera toxin (Invitrogen, Carlsbad, California) was measured in HeLa cells by treating the cells with or without filipin for 1 hour. The cells were then incubated with choleratoxin for 30 minutes on ice with or without filipin, and then allowed to internalize cholera toxin for 1 hour at 37°C. After the internalization the cells were washed in ice-cold PBS and then with an ice-cold acidic buffer (100 mM NaCl, 50 mM glycine, pH 2.5) to remove cholera toxin from the cell surface. The cells were then washed in ice-cold PBS, fixed and the cholera toxin uptake was analyzed by flow cytometry.

### Internalization of ^125^I-EGF

HeLa cells were incubated with 1 nM of ^125^I-EGF (Perkin Elmer, Waltham, Massachusetts) and 9 nM EGF in DMEM with 0.2% BSA for 15 minutes. The cells were washed three times with ice cold PBS before surface-bound ^125^I-EGF was removed by incubating the cells with acetic buffer (0.2 M acetic acid, 0.5 M NaCl, pH 2.5) for 5 minutes on ice followed by a wash with the same buffer. The radioactivity released from the cell surface was subsequently measured in a γ-counter. The cells were hydrolyzed with 1 M NaOH on ice for 30 minutes before the internalized ^125^I-EGF was measured in a γ-counter. Internalized ^125^I-EGF was measured as the percentage of internalized to total cpm.

### Internalization of ^125^I-transferrin

HeLa cells treated with siRNA were incubated with 10 µg/ml ^125^I-transferrin (Perkin Elmer, Waltham, Massachusetts) in DMEM with 0.2% BSA for 15 minutes. The cells were washed three times with ice-cold PBS before being incubated with 3 mg/ml Pronase E in DMEM for 45 minutes on ice. The cell suspension was transferred to Eppendorf tubes and centrifuged at 16000×*g* for 5 minutes at 4°C. The supernatant fraction was removed and counted in a γ-counter. The pellet fraction was washed in cold PBS before analysis by γ-counting. Internalized ^125^I-transferrin was measured as percentage of cpm in the pellet to the total cpm.

### RhoA Inhibition

To inhibit RhoA HeLa cells were incubated for 2.5 hours with 1 µg/ml C3 transferase (Cytoskeleton, Denver, CO, USA) in starvation medium. To test the C3 transferase activity actin fiber integrity was determined after treating HeLa cells with C3 transferase.

### EGFR Degradation Analysis Using ELISA Detection

HeLa cells were incubated with ligands for 0–3 hours in starvation medium at 37°C. Cells were rinsed in cold PBS and scraped off in RIPA lysis buffer (1% NP40, 20 mM MOPS, 0.1% SDS, 1% Na-deoxycholate, 150 mM NaCl, and 1 mM ethylenediaminetetraacetic acid) supplemented with Protease Inhibitor Cocktail Set II and Phosphatase Inhibitor Cocktail Set III (Calbiochem). Cell debris was removed by centrifugation. EGFR levels were then measured using an EGFR ELISA kit (RnD Systems, Minneapolis, Minnesota).

### Microscopy

HeLa cells were seeded in CC2-coated chamber slides. The days after seeding, cells were incubated with ligands in HEPES-buffered DMEM containing 0.2% BSA. Cells were rinsed in cold PBS and fixed in 2% paraformaldehyde. After fixation, cells were permeabilized and blocked in PBS containing 5% normal goat serum (In Vitro) and 0.2% saponin. For visualizing EGFR endocytosis immunofluorescence labeling was performed using primary antibodies (mouse monoclonal anti-EGFR IgG2A antibody (clone 199.12, Thermo Fisher Scientific), mouse monoclonal anti-EEA1 IgG1 antibody (clone 14, BD Biosciences)) and isotype specific secondary antibodies (goat anti-mouse IgG1, and goat anti-mouse IgG2A antibodies) conjugated to alexa488 or alexa568 (Invitrogen, Carlsbad, California). To visualize actin filaments in C3 transferase treated cells phalloidin conjugated to alexa568 (Invitrogen, Carlsbad, California) was used. To visualize macropinocytosis of dextran, 70 kDa lysine-fixable dextran conjugated to alexa488 was applied (Invitrogen, Carlsbad, California).

Microscopy was done using a Zeiss LSM 510 Meta confocal microscope, equipped with 488 and 543 nm excitation lasers, using a 63×1.4 NA oil immersion apochromat objective.

### Image Processing and Quantitative Analysis

Images were sectioned into smaller images containing a single cell each using ImageJ with the MBF plug-in compilation. A binary mask defining the endosomes of the cells was made using Gaussian blur filtering (sigma = 1.0) of the EEA1 channel, and then 8-bit Otsu thresholding. The density of the EGFR signal was then integrated for the endocytic mask as well as for the entire cell. The relative amount of EGFR signal contained within the endocytic mask was calculated by dividing the signal from the mask with the total EGFR signal.

### Statistical Analysis

All statistical analysis was performed using the Graphpad Prism software.

## Supporting Information

Figure S1 **EGFR internalization after RhoA inhibition with C3 transferase.** A: Cells treated with or without C3 transferase were incubated with 10 nM ligand for 15 minutes at 37°C. The amount of cell surface EGFR was determined by flow cytometry and data normalized to unstimulated cells that had not been treated with C3 transferase. Data points represent mean+SEM. Statistical analysis comparing C3 transferase to control treatment for each ligand was performed using two-way ANOVA with Bonferroni posttest. ns = non significant. B: Cells treated with or without C3 transferase were incubated with ^125^I-EGF for 15 minutes at 37°C, the ligand bound on the surface was separated from the internalized. Data points represent mean+SEM. Statistical analysis comparing C3 transferase to control treatment was performed using t-test. ns = non significant. C: Cells treated with or without C3 transferase were labeled with alexa568-conjugated phalloidin and actin fiber integrity was visualized by microscopy. D: Test of EGFR levels. Cells were lysed in RIPA buffer and resolved by SDS-PAGE and western blotting. Actin is used as a loading control.
(TIF)

Figure S2 **Internalization after flotillin1/2 knockdown.** A: Knockdown cells were incubated with 10 nM ligand for 15 minutes at 37°C. The amount of cell surface EGFR was determined by flow cytometry and data normalized to unstimulated cells. Data points represent mean+SEM. Statistical analysis comparing flotillin1/2 siRNA treated cells to mock treatment for each ligand was performed using two-way ANOVA with Bonferroni posttest. ns = non significant. B: Test of the flotillin1/2 knockdown and EGFR levels. Cells were lysed in RIPA buffer and resolved by SDS-PAGE and western blotting. Actin is used as a loading control.
(TIF)

Figure S3 **Internalization after induction of dominant negative Arf6 Q67L mutant.** A: Arf6 Q67L expression was induced by removal of tetracycline from the medium for 48 hours. Cells were incubated with 10 nM ligand for 15 minutes at 37°C. The amount of cell surface EGFR was determined by flow cytometry and data normalized to unstimulated cells. Data points represent mean+SEM. Statistical analysis comparing Arf6 DN to control treatment for each ligand was performed using two-way ANOVA with Bonferroni posttest. ns = non significant. B: Arf6 Q67L expression was induced by removal of tetracycline from the medium for 48 hours. Cells were lysed in RIPA buffer and resolved by SDS-PAGE and western blotting. Actin is used as a loading control.
(TIF)
