# Supplementary figures and images for: Internalization Mechanisms of the Epidermal Growth Factor Receptor after Activation with Different Ligands
Source: PLoS One. 2013 Mar 5;8(3):e58148. doi: 10.1371/journal.pone.0058148 (PMC3589378; doi:10.1371/journal.pone.0058148)

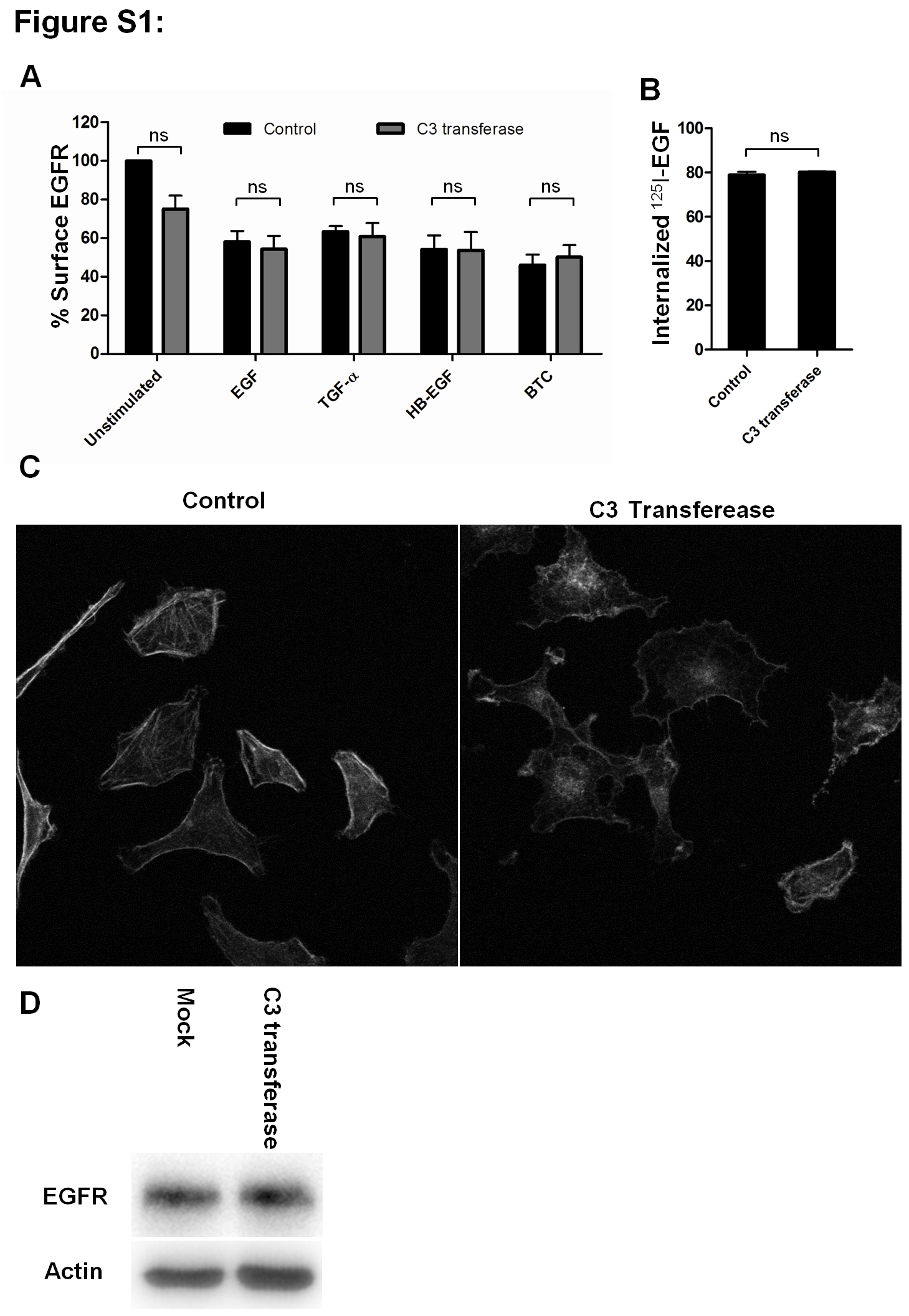

Supplement: Figure S1 — EGFR internalization after RhoA inhibition with C3 transferase. A: Cells treated with or without C3 transferase were incubated with 10 nM ligand for 15 minutes at 37°C. The amount of cell surface EGFR was determined by flow cytometry and data normalized to unstimulated cells that had not been treated with C3 transferase. Data points represent mean+SEM. Statistical analysis comparing C3 transferase to control treatment for each ligand was performed using two-way ANOVA with Bonferroni posttest. ns = non significant. B: Cells treated with or without C3 transferase were incubated with 125I-EGF for 15 minutes at 37°C, the ligand bound on the surface was separated from the internalized. Data points represent mean+SEM. Statistical analysis comparing C3 transferase to control treatment was performed using t-test. ns = non significant. C: Cells treated with or without C3 transferase were labeled with alexa568-conjugated phalloidin and actin fiber integrity was visualized by microscopy. D: Test of EGFR levels. Cells were lysed in RIPA buffer and resolved by SDS-PAGE and western blotting. Actin is used as a loading control. (TIF) [file pone.0058148.s001.tif]

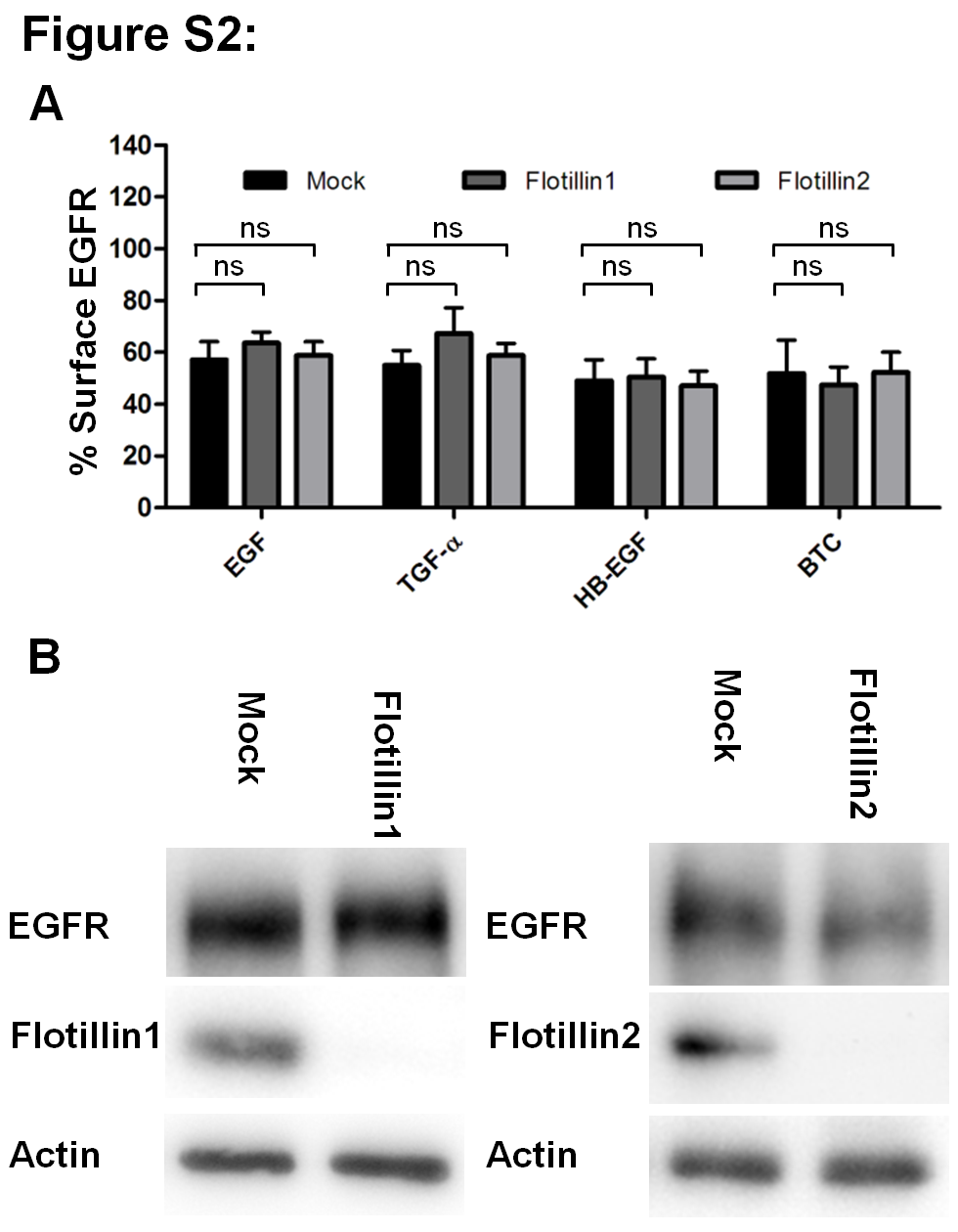

Supplement: Figure S2 — Internalization after flotillin1/2 knockdown. A: Knockdown cells were incubated with 10 nM ligand for 15 minutes at 37°C. The amount of cell surface EGFR was determined by flow cytometry and data normalized to unstimulated cells. Data points represent mean+SEM. Statistical analysis comparing flotillin1/2 siRNA treated cells to mock treatment for each ligand was performed using two-way ANOVA with Bonferroni posttest. ns = non significant. B: Test of the flotillin1/2 knockdown and EGFR levels. Cells were lysed in RIPA buffer and resolved by SDS-PAGE and western blotting. Actin is used as a loading control. (TIF) [file pone.0058148.s002.tif]

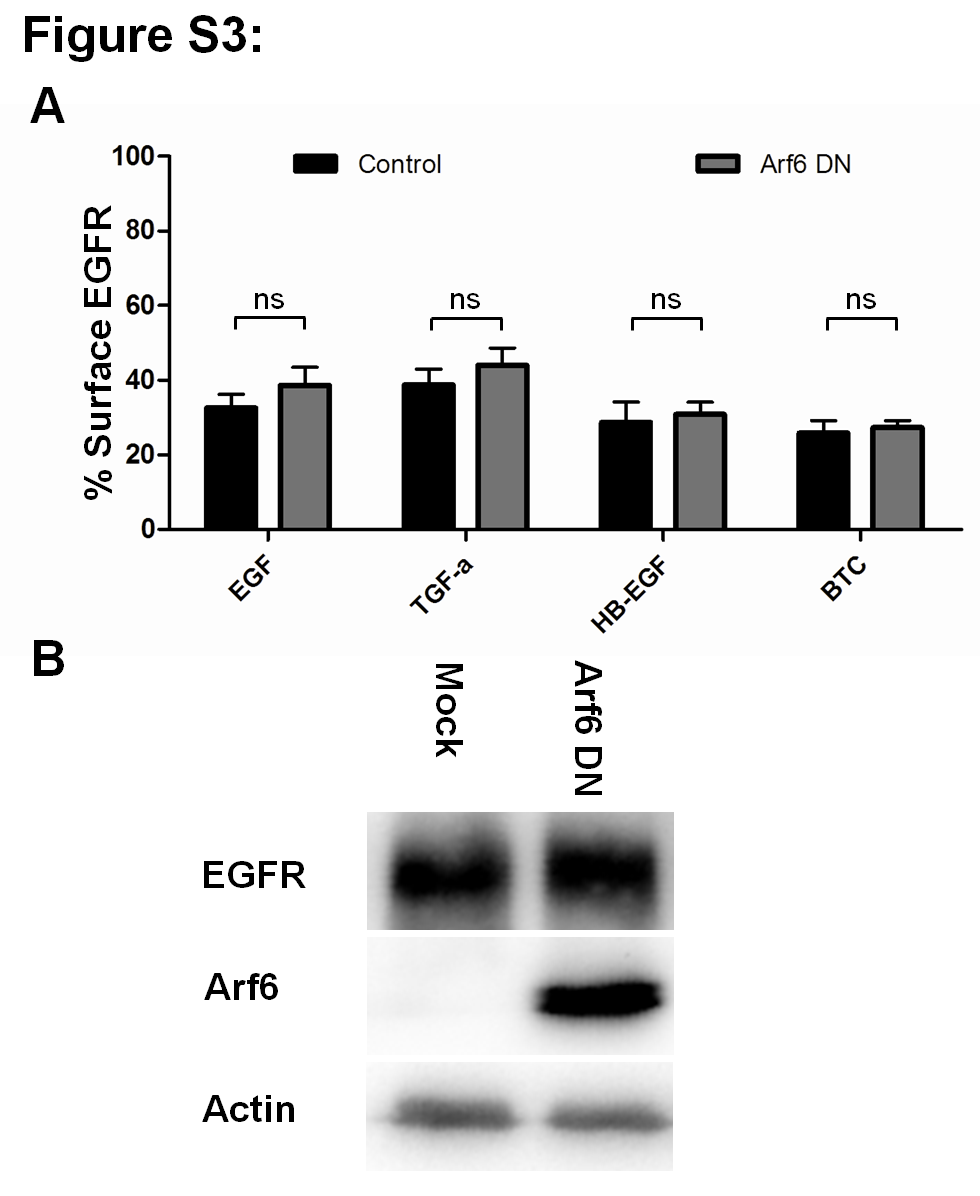

Supplement: Figure S3 — Internalization after induction of dominant negative Arf6 Q67L mutant. A: Arf6 Q67L expression was induced by removal of tetracycline from the medium for 48 hours. Cells were incubated with 10 nM ligand for 15 minutes at 37°C. The amount of cell surface EGFR was determined by flow cytometry and data normalized to unstimulated cells. Data points represent mean+SEM. Statistical analysis comparing Arf6 DN to control treatment for each ligand was performed using two-way ANOVA with Bonferroni posttest. ns = non significant. B: Arf6 Q67L expression was induced by removal of tetracycline from the medium for 48 hours. Cells were lysed in RIPA buffer and resolved by SDS-PAGE and western blotting. Actin is used as a loading control. (TIF) [file pone.0058148.s003.tif]
